# The importance of culturally meaningful activity for health benefits among older Korean immigrant living in the United States

**DOI:** 10.3402/qhw.v10.27501

**Published:** 2015-06-16

**Authors:** Junhyoung Kim, May Kim, Areum Han, Seungtae Chin

**Affiliations:** 1 Department of Recreation, Parks, and Leisure Services Administration, Central Michigan University, Mount Pleasant, MI, USA; 2 Department of Physical Education, Korea University, Seoul, South Korea; 3 Department of Physical Education, Dongduk Women's University, Seoul, South Korea; 4 Department of Taekwondo, Dankook University, Cheonan, South Korea

**Keywords:** Activity, culture, immigrants

## Abstract

Research indicates that participation in culturally meaningful activity is beneficial for immigrants’ health and well-being, yet older Korean immigrants struggle with accepting new cultural perspectives, which can negatively affect their health and well-being. Using in-depth interviews, this study was designed to capture the value of culturally meaningful activities for health among older Korean immigrants. Three themes were identified: (a) improved psychological well-being, (b) enhanced positive emotions and feelings, and (c) social connections developed with others. The findings suggest that by engaging in various culturally meaningful activities, older Korean immigrants gain a sense of social, cultural, and psychological significance in life. This study also provided evidence that older Korean immigrants maintain and develop their cultural identity through culturally meaningful activities.

As of 2012, the number of the Korean Americans in the USA was over 1.7 million, representing 9% of the Asian American population (Asian Matters of America). This population has increased by 41% since 2000; in particular, the number of elderly immigrants accounted for approximately 11% of the total aging population and reached 15.5 million (Leach, 2008; Lee & Holm, 2011). This indicates that older Korean immigrants are one of the fastest growing ethnic groups in the US.

During acculturation, older Asian immigrants experience adaptation challenges because of cultural gaps between their own cultural values and those of the host society (Hsu, Davies, & Hansen, 2004; Hwang & Ting, 2008). Such experience also results in a lack of social support from the host society, family conflicts, and racial discrimination (Chung & Epstein, 2014; Dong, Chang, Wong, Wong, & Simon, 2014; Lin, Bryant, Boldero, & Dow, 2014). The background to this experience is that older Asian immigrants strive to maintain their own cultural heritage and preserve their cultural identities, but this may contribute to low acculturation (Kim, Kleiber, & Kropf, 2001).

A growing body of literature provides evidence that by participating in leisure activities, older immigrants reduce negative psychological symptoms and increase psychological benefits, such as positive emotions, psychological well-being, and life satisfaction (Jang & Chiriboga, 2011; Kim, Chun, Heo, Lee, & Han, 2014; Rueggeberg, Wrosch, & Miller, 2012). Some researchers have articulated the types of leisure activities in which immigrants engaged and found that they created and participated in activities associated with their original culture (Day & Cohen, 2000; Iwasaki, Bartlett, Gottlieb, & Hall, 2009; Mannell, 2007; Stack & Iwasaki, 2009; Waites, 2013). These authors concluded that immigrants gained physical, psychological, cultural, and social benefits as a result of their engagement in culture-related activities.

Iwasaki and Bartlett (2006) defined activities associated with a certain ethnicity or race as *culturally meaningful activities*. In their study, Aboriginal peoples with diabetes engaged in culturally meaningful activities, such as dance, arts, music, spirituality, and cultural identity-related activities. As a result, Aboriginal peoples experienced cultural, psychological, and social benefits. Immigrants were also found to have facilitated their acculturation process and have enhanced health and well-being when they engaged in culturally meaningful activities (Iwasaki & Barlett, 2006; Stack & Iwasaki, 2009; Stodolska & Livengood, 2006; Waites, 2013).

Despite the benefits of engaging in culturally meaningful activity, few studies have sought to understand the health benefits of these activities for older immigrants, although the existing research indicates that they are more likely to struggle with accepting new cultural perspectives compared to younger people (Diwan, Jonnalagadda, & Balaswamy, 2004; Hsu et al., 2004). Moreover, no previous study has identified activities that older adults chose to participate in for health benefits.

## Leisure behaviors associated with culture

Older Korean immigrants may display different patterns of leisure involvement and behaviors because a new cultural environment may influence leisure choices and opportunities open to them. This suggests that older Korean immigrants may maintain engagement in leisure activities that they participated in before immigration, and/or pursue novel activities provided by the host society (Stodolska, 2000).

Some studies have demonstrated the role of culturally meaningful activities as a means of coping with stress (Iwasaki & Bartlett, 2006; Stack & Iwasaki, 2009; Stodolska & Livengood, 2006). In a study of Aboriginal Canadians with diabetes, Iwasaki and Bartlett (2006) found that culturally meaningful activities were used as a coping strategy to deal with stressors related to diabetes and racial discrimination. Similarly, Stack and Iwasaki (2009) found that Afghan immigrants were engaged in cultural celebrations and Afghan associations to reduce stress and improve their well-being. Through culturally meaningful activity, they connected with their own ethnic community, family, and friends (Stack & Iwasaki, 2009). Their qualitative narratives suggested that culture-related activities fostered a sense of significance and purpose in life. In addition, they had an opportunity to interact with other ethnic groups, which facilitated their acculturation into the host society.

Stodolska and Livengood (2006) found that Muslim immigrants maintained their cultural values (e.g., family ties and collectivism) and religious affiliations when living in the US. In accordance with Muslim values, they adhered to dress requirements, cross-gender relations, and restrictions on women traveling alone; Western practices that violated their religious values were also avoided. The authors indicate that these immigrants adhered to their own cultural identities while engaging in Western society within the parameters of their cultural and religious beliefs.

Kim, Dattilo, and Heo (2011) noted that elderly Asian immigrants encounter challenges to engagement in various leisure activities because of cultural and ethnic differences, language barriers, and limited social networks. This research offered practical suggestions on how to encourage elderly Asian immigrants to engage in meaningful activities; one of the more effective solutions for elderly Asian immigrants was to provide activities that reflect their cultural values and beliefs.

Kim et al. (2001) found that elderly Korean immigrants who participated in traditional Korean practices and rituals reported a sense of “being Korean” and developed their cultural and ethnic identities accordingly. Through culturally meaningful activities, they were able to express collectivist behaviors and found comfort within their own ethnic group, which may have promoted attachment to their cultural identities.

In summary, the literature suggests that immigrants’ participation in culturally meaningful activities assists them in maintaining their cultural identity as well as acculturating to the host society. In this study, older Korean immigrants were selected for various reasons. First, these immigrants constitute one of the fastest growing ethnic groups in the US and they have a tendency to preserve their cultural identities and establish ethnic boundaries (Lee & Yoon, 2011; Sung, 2001). However, this ethnic group has been insufficiently studied regarding the exploration of health benefits associated with leisure engagement (Kim & Kim, 2013). Moreover, no previous research has examined older Korean immigrants’ perceptions of the value of culturally meaningful activities in relation to the health benefits they may have experienced. Therefore, the study purpose was to ascertain the health benefits of culturally meaningful activities in which older Korean immigrants participated.

## Methods

This study focused on health benefits as a result of culturally meaningful activity engagement among older Korean immigrants. To respond to this inquiry, semi-structured in-depth interviews were conducted to facilitate an understanding of the cultural complexities of the subjective experiences in the context of their involvement in these activities. This approach is most appropriate when researchers have a specific research inquiry among a certain group of individuals, such as ethnic minorities, immigrants, and the homeless (Hesse-Biber & Leavy, 2006; Kim, 2012).

### Participants

A purposive sampling strategy was used to recruit participants related to their experiences that resulted from participation in activities associated with Korean culture among older Korean immigrants. Participants were recruited with the cooperation of the Korean community and senior centers in the Midwestern US. The principle investigator contacted the program directors of community centers to recruit participants. With permission, he posted flyers and delivered recruitment information. The study criteria were that participants should be over 65 years old, had moved to the US from South Korea, and were able to speak Korean or English. Prospective participants responded via emails and phone calls. The investigator provided a document containing the study purpose, issues relating to confidentiality, and the voluntary nature of the research. The university's Institutional Review Board approved these procedures (#39889). Pseudonyms were used for the participants in the study and are referred to in this paper.

Ultimately, 18 older Korean immigrants (10 males and 8 females) participated in this study. Four had experienced the loss of a spouse. Age ranged from 65 to 85 years (*M*=72.2) and their length of stay in the US ranged from 10 to 52 years (*M*=34 years). Theoretical saturation occurred when the investigator interviewed the 17th participant. To confirm saturation, the investigator conducted another interview; no new information or relationships emerged from the data.

### Data collection

The interviews occurred at the place and time most convenient for each participant. Each interview lasted between 60 and 90 min. Semi-structured questions were developed based on previous literature (Iwasaki & Bartlett, 2006; Iwasaki et al., 2009). To collect rich data and observe the leisure benefits associated with culture, a “grand-tour” and “mini-tour” interviewing strategy was used (Spradley, 1979). Previous studies incorporated this interviewing strategy to understand and explore leisure benefits among older adults (Kim & Kim, 2013; Kim, Yamada, Heo, & Han, 2014). Grand-tour questions enabled us to understand participants’ preferred language and leisure involvement: “Do you feel most comfortable speaking in English or Korean?,” “Please tell me about the kinds of things you do for fun or enjoyment in your free time,” and “Do you participate in activities associated with your own culture? If so, what are they?” To ascertain the leisure benefits related to culture, mini-tour questions were asked: “Why do you participate in these culture-related activities?,” “What benefits do you experience when participating in these culture-related activities?,” and “Based on your experiences, what role, if any, have these culture-related activities had in helping you deal with the challenges in your life as an immigrant?” At the end of the interview, the investigator asked for the participants’ demographic information and assigned pseudonyms to all participants to ensure confidentiality.

### Data analysis

This study followed the five-step analytic process for in-depth interviews presented in McCracken (1988). This analytic data analysis helped researchers determine the categories, relationships, and assumptions associated with participants’ particular life experiences (McCracken, 1988).

*Step 1:* Researchers carefully read the data in the transcripts and made brief notations in the margins. At this stage, the main task was to differentiate the important materials in the transcripts from the unimportant ones.

*Step 2:* The observations that occurred in Step 1 were developed into preliminary descriptive categories according to the evidence presented in the transcripts. After reviewing the researchers’ observations in the margin, additional transcripts were examined to check whether these preliminary categories were present in the rest of the data.

*Step 3:* This stage consisted of a thorough examination of the preliminary codes presented by each researcher; here, we sought to identify connections and understand similar and dissimilar patterns.

*Step 4:* Clusters of comments related to the preliminary codes were examined and basic themes were determined.

*Step 5:* The core themes and subthemes were delineated and interpreted to illustrate the key concepts.

### Trustworthiness

This study used the method of member checking (Lincoln & Guba, 1999) to maximize trustworthiness, and ensure data quality and that our interpretations were congruent with those of the participants. Ten participants were involved in this member-checking process. They reviewed a summary of emergent themes and transcripts to confirm that the interpretation accurately reflected their perceptions.

Several researchers suggested back-translation as a method to improve data credibility (Guillemin, Bomardier, & Beaton, 1993; Suh, Kagan, & Strumpf, 2009). In this study, two bilingual researchers who were not part of the research team participated in this back-translation process. The research team sent randomly chosen paragraphs of the translation to two bilingual researchers so that they could perform the back-translation from Korean to English. Via conference call, they agreed that there were no concerns regarding the conceptual meanings and content of the translation.

Finally, rigor was maintained through a series of meetings with an external expert in qualitative research who ensured that the data and interpretations were consistent. This external checking process affirmed the investigators’ logic of interpretation as well as questions that arose regarding themes and issues.

## Findings

Older Korean immigrants shared a variety of leisure experiences related to culturally meaningful activity. Based on participants’ narratives and statements, the activities in which they engaged in and that they found meaningful included Korean traditional games and activities, celebration of Korean holidays and Korean War heroes, teaching the Korean language and culture to others, and creation of cultural events and programs.

Three themes associated with the outcomes of culturally meaningful activity engagement emerged. These were (a) improved psychological well-being, (b) enhanced positive emotions and feelings, and (c) social connections developed with others. These themes indicated that participants gained social, psychological, and cultural benefits from participating in culturally meaningful activities.

### Improved psychological well-being

Improved psychological well-being was the most salient theme identified in the data. All participants mentioned that they had been challenged in adapting to a new culture and had experienced negative psychological symptoms, such as depression, and feelings of loneliness and isolation from their host society. They believed that their engagement in culturally meaningful activities helped them deal with these negative feelings and emotions and, as a result, they experienced greater psychological well-being. According to them, most other participants in these activities were those associated with Korea in some way, such as other older Korean immigrants, adopted individuals from Korea, Korean War veterans, and anyone interested in Korean culture and language. In the context of culturally meaningful activities, they felt that they had created their own world in which they shared with others a language, social norms, culture, and customs related to Korea.

In addition, participants expressed an emotional attachment to their ethnic identities and formed their own ethnic community in which they developed bonds and attachments with each other.

Since immigrating, I always felt left out from family and community because my adult children were busy with their work and I could not drive to go anywhere. Also, I could not speak English at all. I felt so lonely and depressed … Once I became a member of Enoch (Elderly Korean Organization), I was involved in various activities in the Korean community, Korean food festivals, and a Korean language class. I met other elderly Korean immigrants and we had lots of fun together. It is just like a family and I am not lonely anymore …. (Lee, female, 77)

In this manner, Lee invested herself in cultural activities and joined a social support group, which resulted in improved psychological peace and comfort.

Most participants were committed to sharing the Korean culture and language with others, particularly with children of Korean immigrants who were born in the US or who were adopted from South Korea. For example, Jo (female, 70) and Jang (male, 65) volunteered to teach a Korean language class and encouraged learners to acquire cultural knowledge about Korea, such as customs and etiquette. They educated their students about how to interact with other Korean adults in a culturally appropriate manner, including bowing to others, using two hands, and speaking the Korean language. They also celebrated Korean holidays with their students and had rewarding experiences when doing so. They believed that they experienced psychological comfort and peace, and were proud of their cultural identity and ability to share it with others.

Some participants improved their psychological well-being by engaging in Korean cultural events in the community. For example, Chen (female, 66) participated in Korean food festivals to increase the awareness of Korean culture. She mentioned that she received positive comments and feedback from the community for sharing the uniqueness of Korean food with others. Similarly, Kwon-F (female, 65) promoted Korean cultural events—such as traditional games, customs, and holidays—to teach the local community about Korean identity. She mentioned that such cultural activities made her feel appreciated and worthwhile. For example, she provided a Korean calligraphy class to the community with a presentation on the Korean alphabet's structural sophistication. She said that older adults in the community expressed an interest in learning Korean calligraphy and had positive interactions with other participants. As a result, she felt that she experienced psychological well-being.

These examples reveal how the elderly Korean immigrants participating in this study created and provided cultural activities for the community. This involvement promoted their own psychological comfort and reinforced their cultural identities.

### Enhanced positive emotions and feelings

Enhancement of positive emotions and feelings was identified as another salient theme that emerged from the data. All participants mentioned that engaging in culturally meaningful activities had helped them increase positive emotions and feelings because of the meaningful and rewarding experiences of their engagement. They articulated their enhanced feelings and emotions with various expressions, such as “I had lots of fun when I was teaching at the Korean school,” “Organizing Korean food festivals was so enjoyable,” and “I am so excited to work with people who were adopted from Korea.” Some participants experienced positive psychological transformations using their culture as a resource.

Before I engaged in a volunteer activity to help other Korean immigrants, my life was desperate because the only thing I was able to do was to stay home all day. My English was so bad and I could not drive … I thought of myself as useless and that living in here was very boring … I found that helping others was rewarding and enjoyable and I regained excitement in life. Without this work, my life would be going very badly. (Lee, female, 77)

She also mentioned that doing something meaningful enabled her to lead a better life and develop a positive sense of self.

Some participants experienced positive feelings that resulted from social interactions with others when they participated in culturally meaningful activities. Choi (male, 74), who was involved in an organization for Korean immigrant welfare, provided cultural activities for American veterans of the Korean War. He felt that other older Korean immigrants developed meaningful relationships with these veterans when they shared personal experiences with each other. He also experienced positive interpersonal relationships with other Korean immigrants and veterans when he organized cultural activities. He developed close friendships during these activities, which contributed to positive feelings and meaningful experiences.

In a similar manner, Lee-J (male, 80) is a member of a Korean community that helps elderly Korean adults with English language skills. He volunteered to help others as an interpreter in situations, such as going to the hospital and the bank, as well as taking care of visa-related issues.

I am so happy to give something back to other elderly Koreans because I saw that many of them have lots of challenges and struggles related to adapting to a new culture … I help them to translate English into Korean and sometimes teach English as well. I felt so good when I realized that I was able to help others and saw their lives going in a better direction.

By helping other Korean immigrants, his mood was enhanced and he felt worthwhile.

Some participants reported that they gained more confidence and improved their self-esteem by involving themselves in culturally meaningful activities. For example, Kwon (female, 70) described herself as an “internationally disabled person” because of the language barrier between her and non-Korean speakers. She said that she was depressed and had no motivation to do anything because of the stressors involved in acculturation. After she became involved in Enoch, a Korean organization that sponsors cultural events, she felt that her mood and self-esteem improved; she realized that she was doing something meaningful for the host community.

Most female participants shared their personal experiences of being engaged in mainly passive activities, such as watching television and reading books. However, once they gained information on how to participate in a variety of cultural activities, they mentioned that they became more active physically and socially, and were given rich opportunities to engage in more active leisure activities. By acknowledging their contribution to the community through participation in activities they found culturally meaningful, they felt that they had enhanced their self-esteem and sense of self-worth.

In sum, older Korean immigrants gained emotional benefits, such as positive feelings and confidence, through involvement in culturally meaningful activities. Such involvement produced opportunities for participants to develop close relationships with other immigrants and individuals who were associated with Korean society.

### Social connections with others

All participants felt that they had limited opportunities to interact with other ethnic groups and received limited social support from the host community because of language barriers. They believed that involvement in culturally meaningful activities provided rich opportunities to interact with other ethnic groups and acquire social support from the community. For example, Lee-W (male, 65) said that before he was involved in a Korean community organization,he did not have any friends of a different ethnicity or race. He believed that by offering Korean culture-related events and activities, he experienced positive interethnic contact and interactions, and received emotional and social support from the host community. Similarly, Choi (male, 74), who was a volunteer teaching taekwondo and judo, mentioned that he had developed social connections with others and with the wider community. He also said that he had been invited to demonstrate East Asian martial arts and, as a result, had built a strong relationship with the host community.

Most participants mentioned that they had increased their sense of belonging and connectedness with others in the local community. The programs related to Korean culture that they offered were exhibited in the senior center and in public venues. For example, Lee-F (male, 85) who was a retired college professor, had initiated Korean outreach programs in the community to convey Korean culture and customs to others, and to build a partnership with the local government. He said that the Korean community center sought to create unique Korean cultural activities in the community including classes in calligraphy, taekwondo, language, and special events. Kim-Q (male 68), who was also a member of the center, stated that his involvement in a variety of activities enabled him to feel a part of the local community and connect with others.

Culturally meaningful activities provided an environment in which older Korean immigrant participants encountered others with similar interests, and a venue to exchange ideas. Some participants found that they experienced positive intergroup contact and interactions through Korean language classes.

Adopted adults from South Korea wanted to learn Korean culture and language, and they look like Koreans, but the way they speak and behave is totally different. They always said that they were happy to learn their mother country's culture and language, and we have good connections and friendships. I treated them as my adult children, and they are too. I am so happy that we provide this class and activities for them. (Sunjin, female, 68)

She also mentioned that by engaging in these activities, she had experienced positive contact and interactions with other ethnic groups, which resulted in close relationships.

Similarly, Choi (male, 74) invited American veterans of the Korean War to the Korean Senior Center to share information about Korea, enjoy traditional Korean games, and express gratitude to the veterans. He mentioned that despite language barriers, participants enjoyed completing activities together and were able to establish and develop interpersonal relationships. One example he cited was that older Korean immigrants had taught veterans calligraphy and, in return, veterans shared their favorite activities with elderly Korean immigrants.

Based on participants’ personal experiences, it appeared that culturally meaningful activities offered an opportunity for positive interracial interactions and social connections with the community. By engaging in these, participants increased their sense of belonging and connectedness with others.

## Discussion

Participants in this study created and engaged in various activities associated with their own culture, including traditional games and activities, culture-related events, and language classes and programs. By engaging in these activities, they experienced psychological well-being, enhanced positive feelings and emotions, and developed social connections with others and with the community.

Previous research suggested that adaptation challenges prevented immigrants from participating in leisure activities because of language barriers and a lack of leisure resources (Juniu, 2000, 2002; Yu & Berryman, 1996). Rublee and Shaw (1991) suggested that because of language difficulties, immigrants’ participation in leisure activities was restricted to passive, home-oriented, and childcare-related activities. Although older Korean immigrants in this study encountered adaptation challenges, they created and provided a variety of leisure activities associated with their own culture. This suggests that they developed the ability to overcome adaptation challenges through participation in meaningful activities.

Previous studies have also suggested that older East Asian immigrants encounter numerous adaptation challenges related to negative psychological symptoms, such as depression, loneliness, and isolation from others (Lai, 2004; Nicassio, 1983; Weisman et al., 2005). In this study, older Korean immigrants acquired social, psychological, and cultural ties with others through their activities, and gained health benefits from this engagement. This suggests that engagement in culturally meaningful activities helped these participants reduce negative psychological symptoms and increased their ability to cope with adaptation challenges.

In leisure studies, there is evidence that immigrants are acculturated to a host society and gain social, cultural, and psychological benefits to well-being via engagement in culturally meaningful activities (Iwasaki & Bartlett, 2006; Stack & Iwasaki, 2009). In particular, Iwasaki, Coyle, Shank, Messina, and Porter (2013) suggest that certain ethnic groups found cultural “strength” through these activities by practicing their cultural values and beliefs. This study is aligned with Iwasaki and colleagues’ findings that culturally meaningful activities contributed to social, psychological, emotional, and cultural benefits. In particular, older Korean immigrants demonstrated cultural empowerment, and teaching the Korean language and culture has intensified their ethnic identity.

Some research has suggested that Korean immigrants expressed a strong sense of emotional attachment to their cultural identities and exhibited collectivist behaviors through the activities in which they participated (Kim, 2012; Kim et al., 2001). Our research supports findings that older Korean immigrants strive to maintain their cultural identities through shared activities and teaching their cultural identities to others. Older Korean immigrant participants also used culturally meaningful activities to preserve their cultural identities and communicate with individuals in the host community.

According to intergroup contact theory, interactions among individuals of different races and ethnicities lead to positive intergroup contact, such as the formation of cross-group friendships (Pettigrew & Tropp, 2006, 2008). However, other research indicates that some individuals perceive stress in intergroup interactions and thus avoid personal contact with other ethnic groups (Mallett, Wilson, & Gilbert, 2008; Richeson et al., 2007). Leisure activities, as one form of intergroup interaction, may serve as an important vehicle for positive intergroup contact (Kim, 2012). In this study, older Korean immigrants experienced positive contact with other ethnic groups by engaging in culturally meaningful activities. Our research indicates that personally meaningful activity creates an opportunity for positive intergroup contact.

To some extent, these data are reflective of Erikson's final stage of adult development: integrity versus despair. Successful (i.e., generative) resolution of this dialectic is typified by an outward shift in focus and an interest in leaving a (broadly defined) legacy. For the study participants, motivation for social and productive engagement may be based, at least in part, in generativity (Gruenewald, Liao, & Seeman, 2012; Narushima, 2005). These participants demonstrated the value of being useful and contributing to others, which is evidence of generativity. Further research could usefully investigate whether these immigrants were well -positioned to behave in a generative way before being confronted with the challenges of acculturation. Alternately, Erikson assumes some interaction between development and one's environment. Immigration and acculturation challenges may have provoked a need for participants to preserve their Korean heritage and to pass it on to others in the host country.

### Limitations and need for future research

This study recognizes several limitations. First, the generalization of results is limited because this study focused on a small sample of older Korean immigrants. Larger representative samples are needed to further investigate and generalize the relationships between leisure activities and well-being among immigrant populations.

Second, this study was designed to capture the benefits of culturally meaningful activities among older Korean immigrants; negative implications remain unexamined. As the tendency to interact with the same ethnic group can result in low acculturation and negative psychological symptoms, such as depression and loneliness (Kim, 1999; Mui, 2001), future studies should delineate the positive and negative perspectives that occur as a result of the interactions among and between ethnic immigrant groups. Such research could be useful for interventions to identify optimal pathways to acculturation among older adult immigrants.

Although older Korean immigrants may participate in a variety of leisure activities not associated specifically with their own culture, this study explored only the role of culturally meaningful activities. Future studies are needed to understand the leisure benefits of activities unrelated to an immigrant's culture.

Finally, older immigrants from countries other than Korea may have different immigration experiences and participate in different activities. Cross-cultural study of leisure benefits for older immigrants from various cultures would provide insights into differences in how culture is related to psychosocial well-being. Longitudinal research is also needed to depict the trajectory of acculturation challenges among older immigrants. This study did not measure participants’ level of acculturation or explore the relationship between acculturation and leisure activity among older immigrants across time. However, longitudinal examination of this relationship would help identify how leisure activity contributes to acculturation. Older immigrants may express different levels of involvement and immigration experiences depending on the extent of their acculturation.

Overall, the study findings indicate that benefits accrue to the psychosocial well-being of older Korean immigrants who participate in culturally meaningful activities, including those in which they are helping others to acculturate to a new society. It is important for activity professionals to acknowledge the value of culturally meaningful activities among older Korean immigrants and it is necessary to provide them with various recreational programs related to their own culture.
